# Outcome of conservative and surgical treatment of enchondromas and atypical cartilaginous tumors of the long bones: retrospective analysis of 228 patients

**DOI:** 10.1186/s12891-019-2502-7

**Published:** 2019-03-28

**Authors:** Georg W. Omlor, Vera Lohnherr, Jessica Lange, Simone Gantz, Gunhild Mechtersheimer, Christian Merle, Patric Raiss, Joerg Fellenberg, Burkhard Lehner

**Affiliations:** 1 Department of Orthopaedics, Trauma Surgery and Paraplegiology, Heidelberg University Hospital, Schlierbacher Landstrasse 200a, Heidelberg, Germany; 2 Institute of Pathology Heidelberg, University of Heidelberg, Heidelberg, Germany

**Keywords:** Enchondroma, ACT, Chondrosarcoma, Curettage, Bone-cement, Long bones

## Abstract

**Background:**

Sufficient data on outcome of patients with clinically and radiologically aggressive enchondromas and atypical cartilaginous tumors (ACT) is lacking. We therefore analyzed both conservatively and surgically treated patients with lesions, which were not distinguishable between benign enchondroma and low-grade malignant ACT based upon clinical and radiologic appearance.

**Methods:**

The series included 228 consecutive cases with a follow-up > 24 months to assess radiological, histological, and clinical outcome including recurrences and complications. Pain, satisfaction, functional limitations, and the musculoskeletal tumor society (MSTS) score were evaluated to judge both function and emotional acceptance at final follow-up.

**Results:**

Follow-up took place at a mean of 82 (median 75) months. The 228 patients all had comparable clinical and radiological findings. Of these, 153 patients were treated conservatively, while the other 75 patients underwent intralesional curettage. Besides clinical and radiological aggressiveness, most lesions were histologically judged as benign enchondromas. 9 cases were determined to be ACT, while the remaining 7 cases had indeterminate histology. After surgery, three patients developed a recurrence, and a further seven had complications of which six were related to osteosynthesis. Both groups had excellent and almost equal MSTS scores of 96 and 97%, respectively, but significantly less functional limitations were found in the non-surgery group. Further sub-analyses were performed to reduce selection bias. Sub-analysis of histologically diagnosed enchondromas in the surgery group found more pain, less function, and worse MSTS score compared to the non-surgery group. Sub-analysis of smaller lesions (< 4.4 cm) did not show significant differences. In contrast, larger lesions displayed significantly worse results after surgery compared to conservative treatment (enchondromas > 4.4 cm: MSTS score: 94.0% versus 97.3%, *p* = 0.007; pain 2.3 versus 0.8, *p* = 0.001). The majority of lesions treated surgically was filled with polymethylmethacrylate bone-cement, while the remainder was filled with cancellous-bone, without significant difference in clinical outcome.

**Conclusion:**

Feasibility of intralesional curettage strategies for symptomatic benign to low-grade malignant chondrogenic tumors was supported. Surgery, however, did not prove superior compared to conservative clinical and radiological observation. Due to the low risk of transformation into higher-grade tumors and better functional results, more lesions might just be observed if continuous follow-up is assured.

## Background

Enchondromas belong to the most frequently found cartilaginous bone lesions [1, 2]. Most enchondromas are asymptomatic but malignant transformation into secondary chondrosarcoma is possible in 1–9% depending on localization, pain, size, and radiologic presentation [1–5]. Chondrosarcoma grade I is a low-grade malignant tumor characterized by local aggressiveness leading to destruction of cortical bone, increased pain, frequent local recurrences, but usually does not develop metastases; consequently, it was renamed by the WHO to be an ‘atypical cartilaginous tumor’ (ACT) [1, 2, 6]. Several reports discuss metastatic potential with a 2–8% probability depending on localization and recurrence [7, 8]. Histological and radiological differentiation between clinically and radiologically aggressive enchondromas and ACTs is extremely challenging and often not possible, so treatment depends mainly on the clinical behavior without specific differentiation between both entities [1, 4, 9–13]. Treatments include a wide spectrum from wait and see to various surgical strategies without significant evidence and consensus to support the different strategies [4, 14–17]. Lesions with high local aggressiveness and pain are often surgically resected, whereas smaller asymptomatic enchondromas are most often treated conservatively [7, 10]. Nevertheless, there is no evidence if aggressive enchondromas and ACTs should be treated surgically or not. Even for ACTs, conservative strategies with radiographic follow-up are proposed to prevent the overtreatment and morbidity associated with surgery despite being a low-grade malignant tumor [4, 9, 18].

Differences of clinical outcome between conservatively and surgically treated patients are unknown and treatment strategies remain highly individual. Therefore, the study objective was to perform the thorough evaluation of radiological and clinical outcomes in our series of 228 cases including recurrences, metastases, and functional and emotional parameters of both groups to distinguish potential differences and to improve decision making for the treatment of these benign to low-grade malignant bone tumors.

## Materials and methods

All patients diagnosed with either aggressive enchondroma or ACT treated in our university orthopaedic oncology outpatient clinic between 2005 and 2015 were retrieved from a prospectively enrolled database and retrospectively analyzed. Approval was given by the local ethics committee of the University of Heidelberg, Germany. Only aggressive lesions that were suspicious for enchondroma or ACT with the need for continuous follow-up in our oncology department were included. Asymptomatic enchondromas without radiological signs of aggressiveness and without recommended further follow-up through our department were not included. Cases initially suspicious for higher grade II or III chondrosarcomas (extensive cortical bone enlargement, large interruption of the cortex, extensive invasion of the soft tissues) were excluded. Further exclusion criteria were follow-up shorter than 24 months (*n* = 92), and Ollier’s disease (*n* = 16). Hence, 228 consecutive patients could be included. Decision of conservative versus surgical treatment was done individually as clinical and radiologic appearance would have justified both strategies. Retrospective analysis of medical histories did not reveal hard criteria for initial decision making as both groups showed specific local pain and radiological signs of aggressiveness (large lesion sizes; endosteal scalloping with partial destruction of the cortical bone; similar calcification patterns). Analysis of both alternatives with conservative follow-up or immediate surgical treatment had been discussed with the patients. After consideration of individual advantages and disadvantages decision was made together with the patient, as there is still no evidence and consensus in the literature, which lesions should be treated surgically or not [4, 9, 14, 15, 18]. Radiological evaluation with x-rays and MRIs was performed initially and at regular intervals between 6 and 24 months until final follow-up in both conservatively and surgically treated cases. Images were reviewed by a musculoskeletal radiologist sub-specialized in orthopaedic oncology. Radiologic reports were well-documented with descriptions of the chondroid matrix, calcification patterns, and destruction of cortical bone (endosteal scalloping). Due to heterogeneous geometrical configurations of the lesions, the size was evaluated by measuring the longest diameter. CT-scans of the lesions were only performed in limited cases. Pulmonary x-rays and in the cases of histologically diagnosed ACTs an additional pulmonary CT scan was obtained to rule out metastases.

Histological evaluation was performed by the pathologists of our university pathology department. Final diagnosis was confirmed in a consensus decision together with the senior pathologist (GM). The main histological criterion to distinguish ACT from enchondroma was permeative infiltrative tumor growth with encapsulation of lamellar bone trabeculae [19]. Further criteria were hypercellularity, cellular atypia, myxoid areas, and cell necrosis. Clinical evaluation with physical examination was performed upon presentation to our musculoskeletal oncology outpatient clinic, which is responsible for all orthopaedic tumor cases at our university. Patient demographics and clinical histories were retrieved including detailed information on surgical treatment, recurrences, and complications. For systematic evaluation of pain, patient satisfaction, and functional limitation at final follow-up, an additional telephone interview was performed with a standardized questionnaire available for *n* = 59 patients in the surgery group and *n* = 125 patients in the non-surgery group. Analog scale ratings for pain and patient satisfaction from 0 to 10 were obtained. Clinical function was semi-quantitatively evaluated for the affected body part with the oxford knee score [20], oxford hip score [21], foot and ankle disability score [22], or quick disabilities of arm shoulder and hand score [23]. Score values were recalculated into a 4-point grading with a range from 0 to 3 points (0 = no functional deficit; 3 = high disability with poor function). Additionally, outcome was evaluated by the well-established musculoskeletal tumor society (MSTS) score combining functional parameters with emotional acceptance and pain [24].

Descriptive statistics were calculated as mean and range for numerical variables and for frequencies with corresponding percentages for categorical variables. Statistical analysis was performed for the outcome measures “pain”, “satisfaction”, “functional limitations”, “MSTS score”, “lesion size”, “complications”, and “recurrence”. To compare the differences, Student T-tests, Mann-Whitney-U-tests, and Kruskal-Wallis-tests were performed depending on scale level and distribution of the data. Statistical significance was assumed at a *p*-value < 0.05.

## Results

The mean follow-up was 82 (median 75) months with a range from 25 to 266 months for the whole series. Mean (median) follow-up of the 153 conservatively treated cases was 88 (79) months with a range from 25 to 266 months versus 70 (57) months with a range from 26 to 217 months for the 75 surgically treated cases. Localizations, demographics, and treatment strategies are presented in Table 1.

**Table 1:** Patient demographics with localizations and way of treatment

	number	percentage
gender (mean age)		
total (47-years)	228	100
female (47-years)	154	68
male (47-years)	74	32
localization		
femur	112	49
humerus	86	38
tibia	21	9
fibula	8	4
ulna	1	0.4
treatment strategy		
non-surgical	153	67
surgery	75	33
curettage	2	1
curettage + bone-cement	34	15
curettage + bone-cement + osteosynthesis	27	12
curettage + cancellous-bone	6	3
curettage + cancellous-bone + osteosynthesis	6	3

### Surgically treated patients

**Fig. 1:**
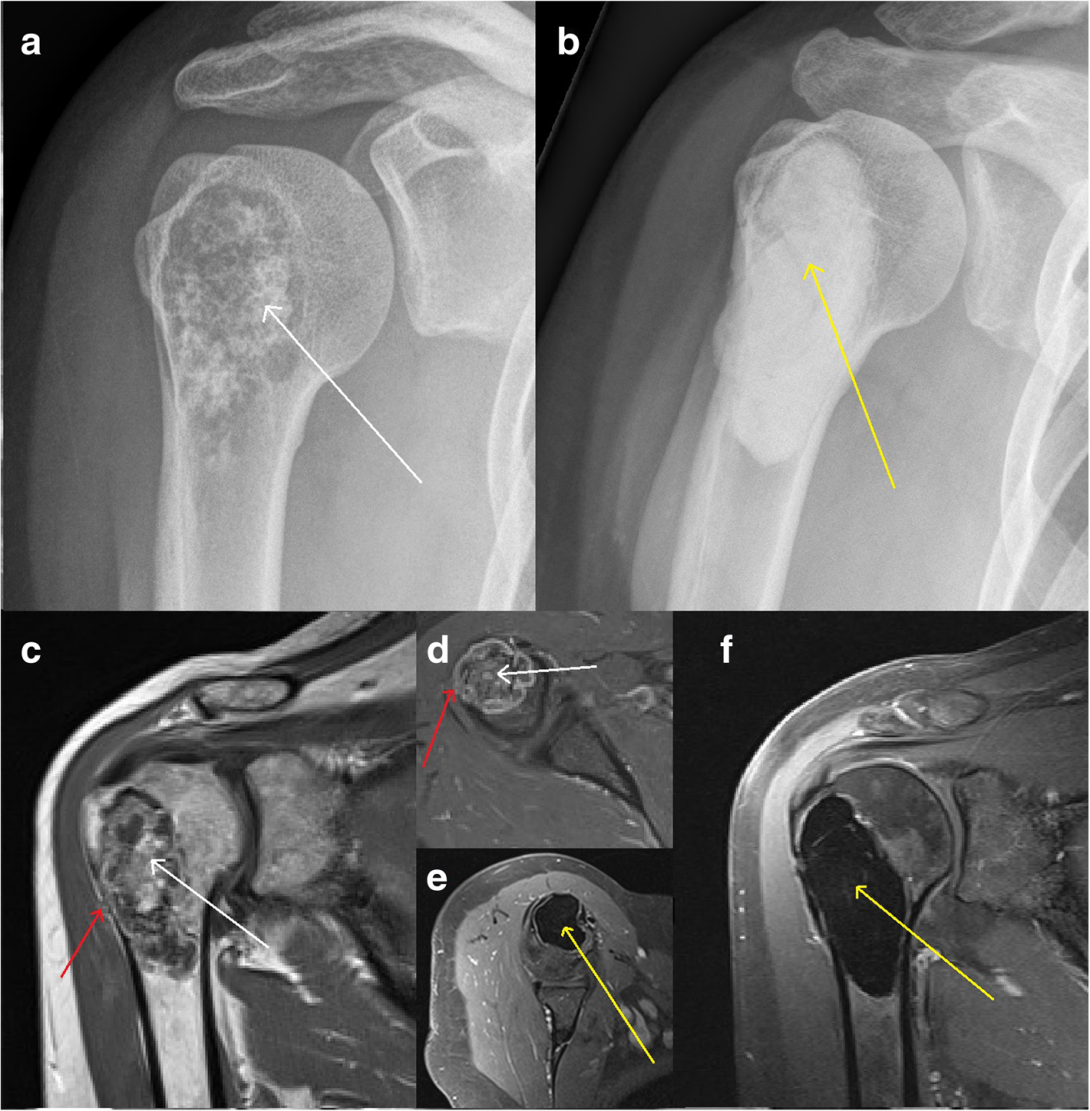
Surgical treatment at the proximal humerus Typical chondroid matrix (white arrows) is visible in ap x-ray (**a**) and coronal (**c**) and axial (**d**) T1 dotarem contrast-enhanced MRI. The decision to perform surgery was made due to local pain, lesion size and radiological signs of aggressiveness with endosteal scalloping (red arrows). After rigorous intralesional curettage and use of a high-speed burr, the lesion was filled with polymethylmethacrylate bone-cement (yellow arrows in **b**, **e**, and **f**).

**Fig. 2:**
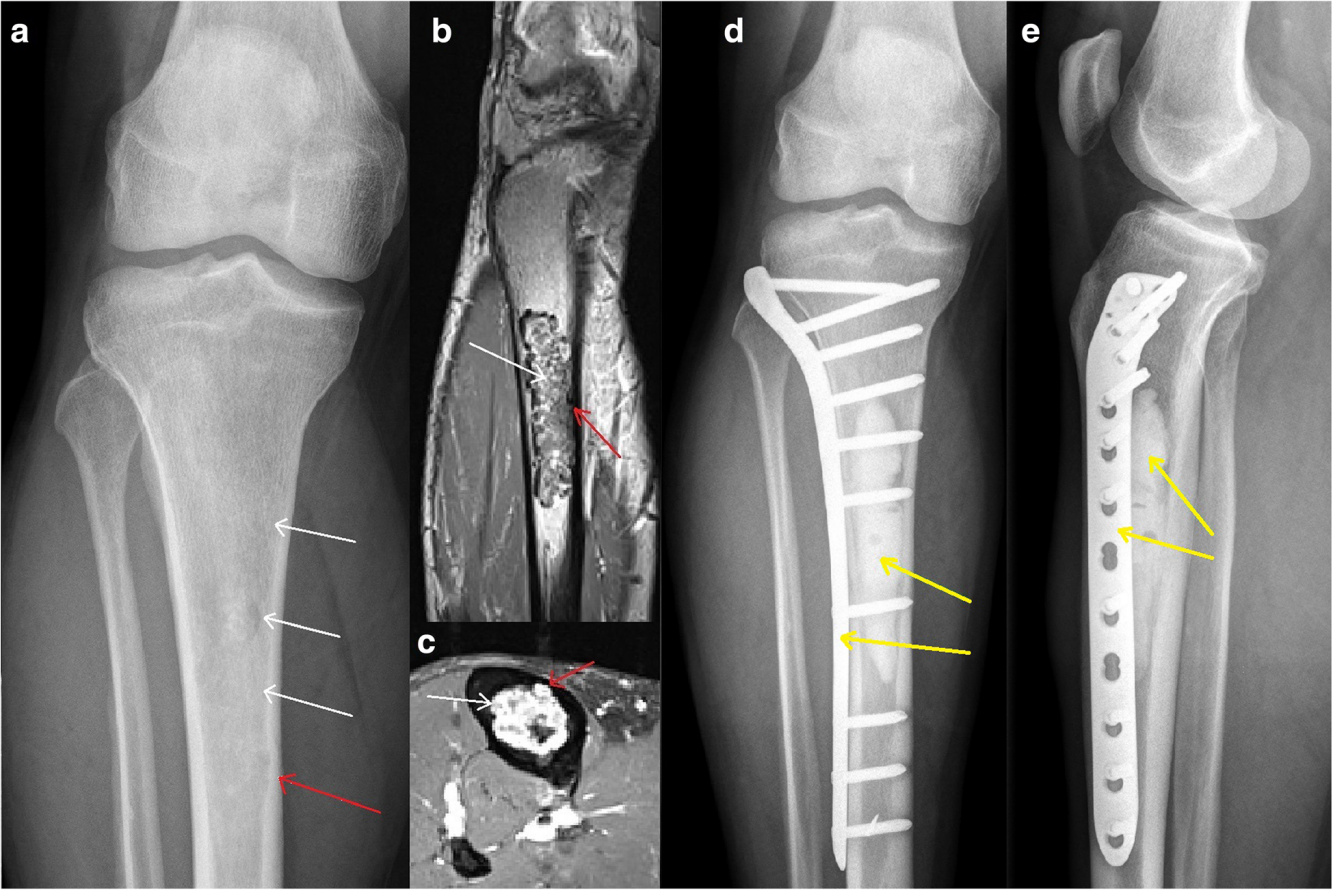
Surgical treatment at the proximal tibia Typical chondroid matrix (white arrows) and endosteal scalloping (red arrows) is visible in ap x-ray (**a**) and coronal (**b**) and axial (**c**) T1 dotarem contrast-enhanced MRI. As a result of the large biomechanical stresses on the lesion location, a compound osteosynthesis using polymethylmethacrylate bone-cement and a plate (yellow arrows in ap (**d**) and lateral (**e**) x-rays) was performed after rigorous curettage and use of a high-speed burr.

Surgery was performed in 75 patients with extensive intralesional curettage and additional use of a high-speed burr. In 39 of these cases, additional H_2_O_2_ application was documented to cleanse the curetted lesion and reduce the risk for recurrence. Surgical strategies could be divided into 5 sub-groups with different frequencies depicted in Table 1. Most lesions were filled with polymethylmethacrylate bone-cement (Fig. 1) providing immediate stability and destruction of potentially remaining tumor cells at the margins due to the exothermic polymerization process when the bone-cement hardens. Additional osteosynthesis (compound bone-cement-plate-osteosynthesis) was used to increase stability (Fig. 2).

Most surgically excised lesions (79%; *n* = 59) were histologically described as benign enchondromas in the final histological analyses. ACT was only diagnosed in 12% (*n* = 9) of surgically treated patients, and in a further 9% (*n* = 7) histology was not distinguishable between enchondroma and ACT.

Open surgical incision biopsy was only performed in 20 cases due to ambiguous radiological appearance. Of those, enchondroma was diagnosed in 15 cases, ACT in 4 cases, and in 1 case histology was not distinguishable between enchondroma and ACT. The biopsied patients were either radiologically followed (*n* = 7) or underwent excision surgery (*n* = 13; *n* = 6 excision + bone-cement + osteosynthesis; *n* = 5 excision + bone-cement; *n* = 2 excision + cancellous-bone + osteosynthesis). In all these cases, final histology confirmed initial diagnosis from incision biopsy. The remaining 62 surgically treated cases received initial intralesional curettage without prior biopsy. Incisional biopsy was avoided due to sufficient radiologic diagnosis and to rule out sampling error in histological evaluation and to spare a second approach. No surgically treated patient was lost to follow-up.

### Conservatively treated patients

**Fig. 3:**
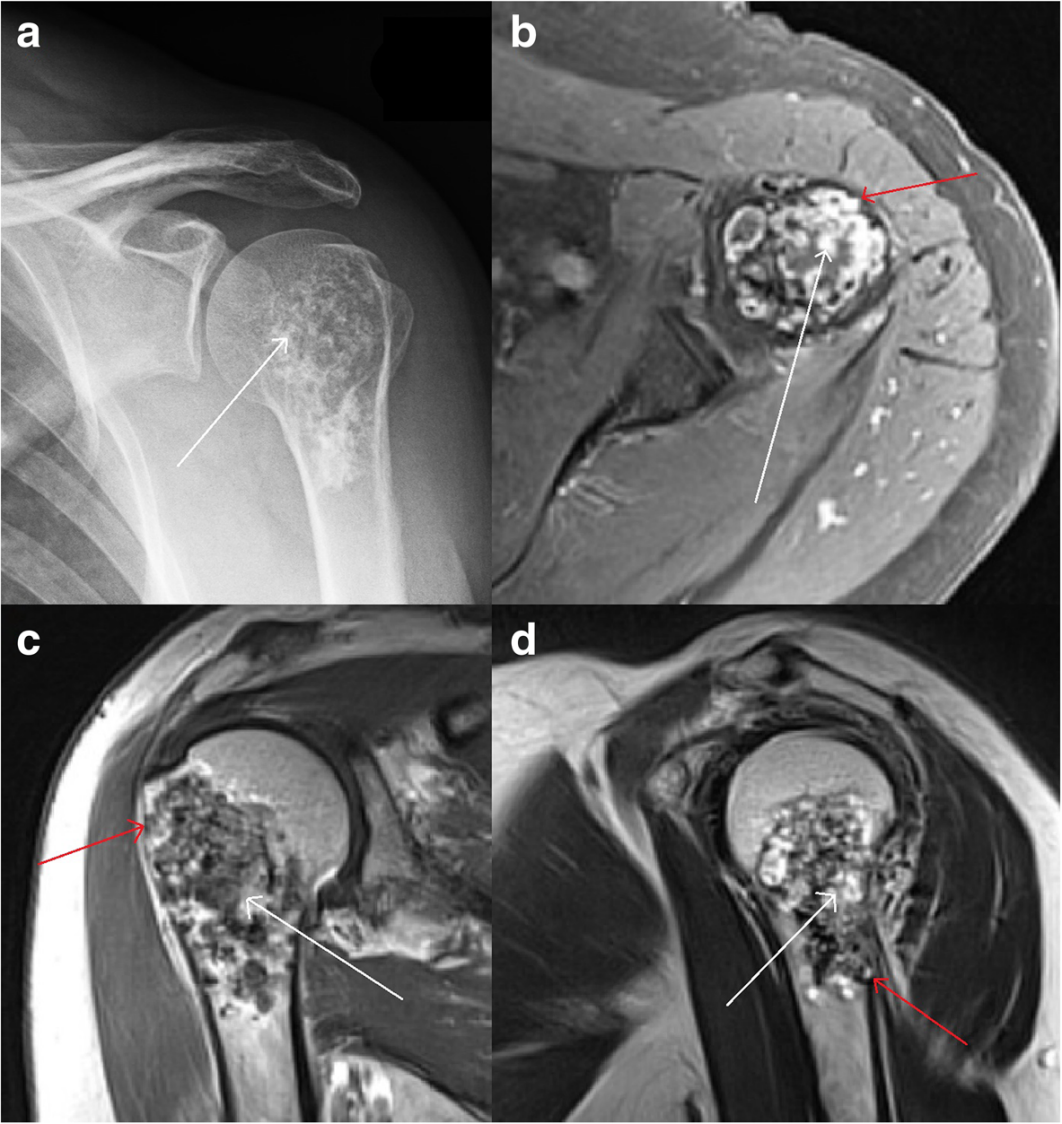
Conservative treatment at the proximal humerus Typical chondroid matrix (white arrows) is visible in ap X-ray (**a**) and axial (**b**), coronal (**c**), and sagittal (**d**) T1 dotarem contrast-enhanced MRI. Local pain, size and radiological signs of aggressiveness with endosteal scalloping (red arrows) caused presentation and follow-up in our orthopaedic oncology department. The patient declined surgical excision, and was regularly followed-up with MRI and x-ray to rule out significant tumor growth or increase in endosteal scalloping.

**Fig. 4:**
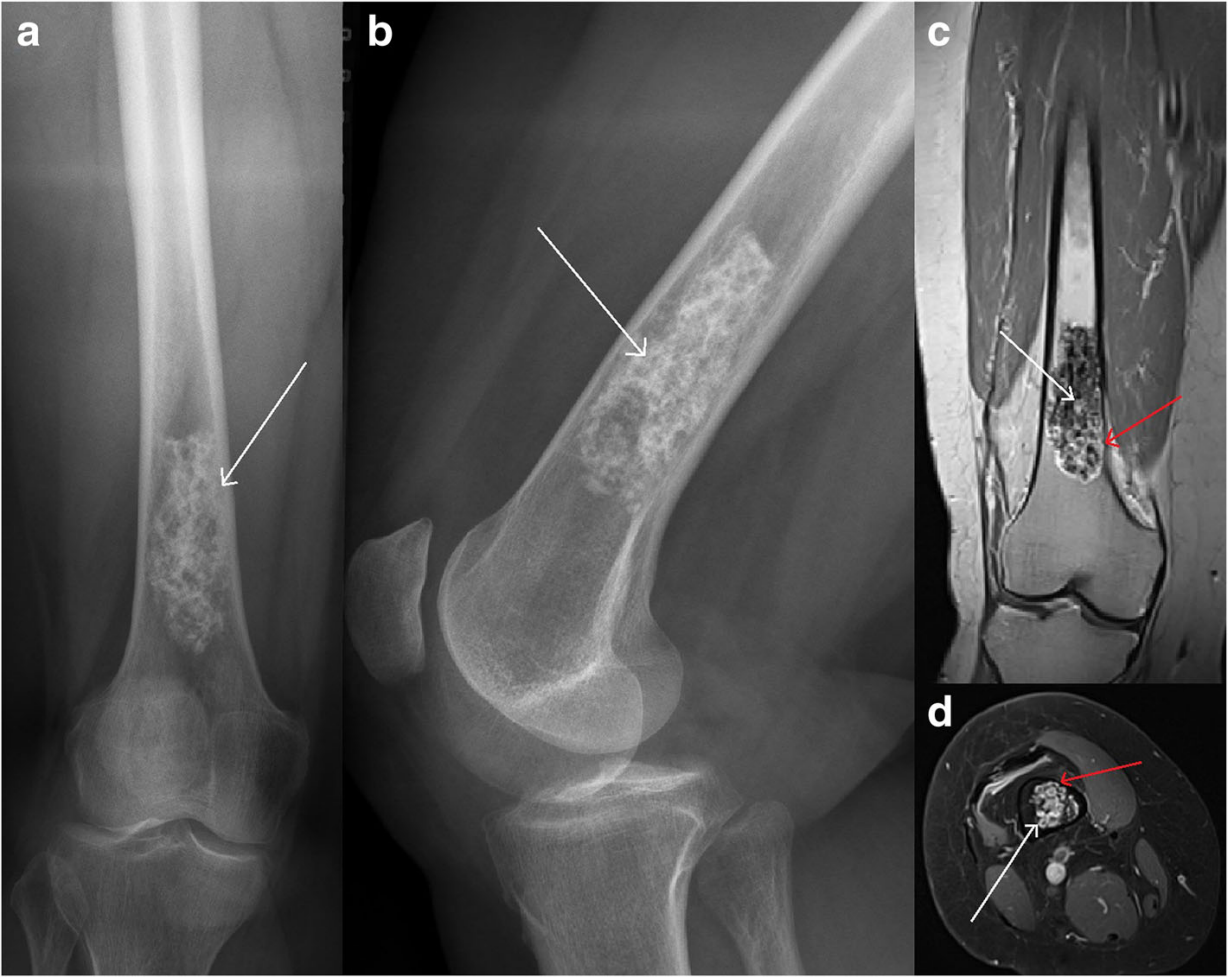
Conservative treatment at the distal femur Typical chondroid matrix (white arrows) is visible in ap (**a**) and lateral (**b**) X-rays and coronal (**c**) and axial (**d**) T1 dotarem contrast-enhanced MRI. Despite local pain, large lesion size, and endosteal scalloping (red arrows) surgery was not performed.

The other 153 patients did not receive surgery but had similar clinical and radiological findings as well as equal mean age and demographics as surgically treated patients (Table 1). Lesions were defined as clinically and radiologically aggressive enchondromas (Figs. 3 and 4). As surgical treatment was not performed, regular follow-up was recommended and carried out in our orthopaedic oncology outpatient clinic. Conservatively followed lesions remained qualitatively unchanged during radiologic follow-up without significant increase of endosteal scalloping or instability. None of the conservatively treated patients was lost to follow-up.

### Lesion size

**Fig. 5:**
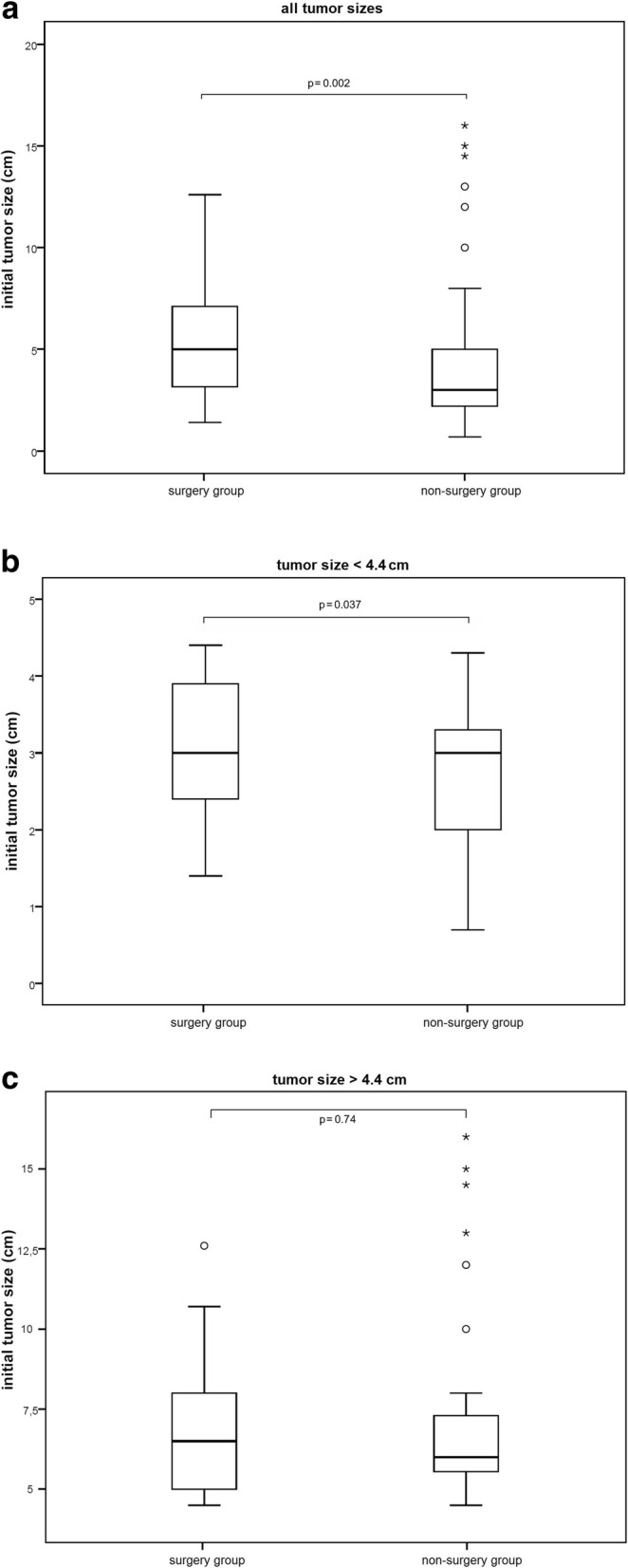
Box plots comparing lesion sizes of the surgery and the non-surgery group for the whole collective including all lesion sizes (**a**) and for lesions with tumor sizes < 4.4 cm (**b**) or > 4.4 cm (**c**)

Mean lesion size of all cases was 4.4 ± 2.8 cm. Sizes of the surgery and non-surgery group are depicted in Tables 2 and 3 and in Fig. 5. Conservatively treated patients showed a trend (*p* = 0.09) towards minimal (1%) increase of radiological lesion size during follow-up.

**Table 2:** Lesion sizes depending on histological diagnosis

surgery group	initial tumor size mean in cm (±SD)
all (*n* = 75)	5.2 (±2.6)
histology (*p* = 0.7)	
enchondroma (*n* = 59)	5.2 (±2.7)
ACT (*n* = 9)	5.7 (±2.5)
enchondroma or ACT (*n* = 7)	5.1 (±2.6)

**Table 3:** Lesion sizes of surgically and conservatively treated tumors

	surgery group initial tumor size mean in cm (±SD)	non-surgery group initial tumor size mean in cm (±SD)	significance (*p*-value)
all (*n* = 228)	5.2 (±2.6)	4.0 (±2.8)	*p* = 0.002
localization			
upper limb (*n* = 87)	5.2 (±2.5)	4.7 (±3.1)	*p* = 0.4
hip region (*n* = 31)	5.6 (±2.8)	4.0 (±2.4)	*p* = 0.2
knee region (*n* = 105)	5.2 (±2.7)	3.6 (±2.6)	*p* = 0.01
ankle region (*n* = 5)	4.9 (±3.4)	4.0 (±0.0)	*p* = 0.8

Comparison of the lesion sizes of the surgical group with the non-surgery group showed that for the whole collective mean lesion size of surgically treated lesions was slightly larger (5.2 ± 2.6 cm versus 4.0 ± 2.8 cm; (*p* = 0.002) (Fig. 5a). Sub-analysis of lesions larger or smaller than the mean size of 4.4 cm was additionally performed. Lesions < 4.4 cm (Fig. 5b) showed a slightly larger mean lesion size in the surgery group (*p* = 0.037), whereas lesions > 4.4 cm (Fig. 5c) did not show significant size differences in the surgery and non-surgery group (*p* = 0.74). Hence, comparison of these larger lesions > 4.4 cm will offer best comparability with least possible selection bias for both groups.

Comparison of lesion sizes of different localizations within the surgery or non-surgery group did not show statistically significant differences, neither in the surgical group (*p* = 0.9), nor in the non-surgery group (*p* = 0.2). Sizes of lesions with different histology were also not significantly different (Table 2).

Considering different localizations in both groups, lesion size was only significantly larger for lesions in the knee region, whereas there were no significant differences in lesions of the upper limb, the hip region and the ankle region between surgically and conservatively treated cases of the whole collective (Table 3).

### Lesion characteristics

Significant differences of the aggressiveness of the lesions treated surgically or conservatively were not detectable. All lesions showed at least some cortical scalloping, but quantification was not possible due to highly heterogeneous appearance and localizations. The pattern of calcification was also highly variable. Interestingly, lesions with final histological diagnosis of ACT did not show more scalloping nor more inhomogeneous calcification patterns.

### Recurrences

Three surgically treated patients showed recurrence. One recurrence was found in a case with a lesion size < 4.4 cm (1.5 cm) compared to 2 recurrences in cases with a lesion size > 4.4 cm (5.5-6 cm). One patient had recurrence at the proximal humerus 4 years after curettage. He received revision surgery with implantation of bone-cement. Histology confirmed enchondroma recurrence. The 2nd case with early enchondroma recurrence within one year received re-curettage and no further recurrence was found. Another patient with initial biopsy at the proximal tibia and histological diagnosis of enchondroma did not receive curettage. 6 years later, however, the lesion was growing and radiologically suspicious. Curettage with implantation of bone-cement confirmed ACT in final histology. Another 2 years later, contrast MRI revealed recurrence and hence revision-surgery was performed with again intralesional curettage and re-implantation of bone-cement. Histology confirmed ACT again. Until final follow-up, no further recurrence was found.

### Complications

Seven out of 75 surgically treated patients had postoperative fractures or revisions due to intra-articular screw position (Table 4). Other complications (infection, thrombosis, hematoma) were not noted. Lesions with complications were not significantly larger (mean size 5.8 cm). Three out of 7 complications were found in cases with a lesion size < 4.4 cm (3.5–3.8 cm) compared to 4 complications in lesions with a size > 4.4 cm (5.0–10.7 cm). Higher complication rate after osteosynthesis was statistically significant (*p* = 0.01). Patients without surgery had no fractures or other complications.

**Table 4:** Complications after surgical treatment

	fracture	intra-articular screw
**total** (*n* = 75)	5	2
Localization		
upper limb	1	1
hip region	1	0
knee region	3	1
ankle region	0	0
histology		
enchondroma	4	2
enchondroma or ACT	1	0
surgical strategy		
curettage + bone-cement	1	0
curettage + bone-cement + osteosynthesis	4	2

### Clinical outcome

All patients showed excellent clinical outcomes, no matter whether treated surgically or conservatively with an MSTS score of 96% (mean 28.8 ± 1.6) and 97% (mean 29.2 ± 1.3), respectively (*p* = 0.13). For lesions < 4.4 cm the MSTS score was 29.3 ± 1.3 and 29.2 ± 1.3, respectively (*p* = 0.61). For lesions > 4.4 cm, the MSTS score was significantly lower in surgically treated patients (28.4 ± 1.7 and 29.2 ± 1.5, respectively; *p* = 0.02). Surgically treated patients had significantly more functional limitations with a mean score value of 0.2 ± 0.5 versus 0.1 ± 0.3 in the non-surgery group (*p* = 0.03) but considering different lesion sizes statistical significance was lost (lesions < 4.4 cm: 0.2 ± 0.5 and 0.1 ± 0.3, respectively (*p* = 0.14); lesions > 4.4 cm 0.2 ± 0.6 and 0.1 ± 0.4, respectively (p = 0.14). Overall patient satisfaction did not show significant differences between the surgery and the non-surgery group (whole collective: 9.0 ± 1.4 versus 9.2 ± 1.2, *p* = 0.39; lesions < 4.4 cm: 9.2 ± 1.3 versus 9.2 ± 1.2, *p* = 0.98; lesions > 4.4 cm: 8.8 ± 1.5 versus 9.2 ± 1.3, *p* = 0.35). For those patients with final histological diagnosis of enchondroma excluding ACT in the surgery group, differences of clinical outcome were even more significant (Tables 5, 6 and 7). Comparison of different surgical treatment modalities did not reveal significant differences in clinical outcome between bone-cement filling and cancellous-bone filling. Surgery involving adjunctive osteosynthesis showed worse patient satisfaction compared to treatment without osteosynthesis (mean 8.6 ± 1.5 versus 9.3 ± 1.3; p = 0.03), more pain (mean 2.4 ± 2.0 versus 0.6 ± 1.4; *p* < 0.0001), and a lower MSTS score of 94% vs. 98% (mean 28.1 ± 1.6 versus 29.3 ± 1.3; *p* = 0.001).

**Table 5:** Clinical outcome of enchondromas treated surgically or conservatively without consideration of lesion size. Clinical score values are shown as mean with standard deviation (±SD)

	surgery group enchondromas (*n* = 51) (histologically diagnosed)	non-surgery group enchondromas (*n* = 125) (radiologically diagnosed)	significance (*p*-value)
functional limitation score			
rating scale 0–3	0.2 (±0.5)	0.1 (±0.3)	0.036
pain score			
rating scale 0–10	1.6 (±2.1)	1.0 (±1.7)	0.024
satisfaction score			
rating scale 0–10	8.8 (±1.5)	9.2 (±1.2)	0.057
MSTS score			
rating scale 0–30	28.6 (±1.7)	29.2 (±1.3)	0.018

**Table 6:** Sub-group with lesion size < 4.4 cm: clinical outcome of enchondromas treated surgically or conservatively. Clinical score values are shown as mean with standard deviation (±SD)

	surgery group enchondromas (*n* = 27) (histologically diagnosed)	non-surgery group enchondromas (*n* = 87) (radiologically diagnosed)	significance (*p*-value)
functional limitation score			
rating scale 0–3	0.2 (±0.5)	0.1 (±0.3)	0.20
pain score			
rating scale 0–10	0.7 (±1.4)	1.1 (±1.8)	0.48
satisfaction score			
rating scale 0–10	9.1 (±1.4)	9.2 (±1.2)	0.84
MSTS score			
rating scale 0–30	29.2 (±1.3)	29.2 (±1.3)	0.87

**Table 7:** Sub-group with lesion size > 4.4 cm: clinical outcome of enchondromas treated surgically or conservatively. Clinical score values are shown as mean with standard deviation (±SD)

	surgery group enchondromas (*n* = 24) (histologically diagnosed)	non-surgery group enchondromas (*n* = 38) (radiologically diagnosed)	significance (p-value)
functional limitation score			
rating scale 0–3	0.3 (±0.6)	0.1 (±0.4)	0.06
pain score			
rating scale 0–10	2.3 (±2.1)	0.8 (±1.7)	0.001
satisfaction score			
rating scale 0–10	8.7 (±1.6)	9.2 (±1.3)	0.18
MSTS score			
rating scale 0–30	28.2 (±1.7)	29.2 (±1.5)	0.007

## Discussion

We analyzed the outcome after both surgical and conservative treatment of clinically and radiologically aggressive benign to low-grade malignant chondrogenic tumors of the long bones of the upper and lower extremities. The surgically treated cases were evaluated histologically, and diagnosed as benign enchondromas in the majority of the cases (*n* = 59), while low-grade malignant ACT was diagnosed in only a few cases (*n* = 9); in the remaining 7 cases, the histology was unable to differentiate between ACT and enchondroma, resulting from the known difficulties in histologically differentiating both entities [1, 9, 10, 13, 19]. Comparison of all conservatively and surgically treated lesions showed excellent clinical results, however, the functional limitations were increased after surgical treatment. Surgically treated patients with histological diagnosis of enchondroma showed worse results for functional limitations, pain, and the MSTS score compared to conservatively treated patients. Sub-analysis of cases with larger tumor size (> 4.4 cm) displayed significantly worse clinical results with regard to pain and the MSTS score in the surgery group compared to the conservative cohort. For surgically treated patients, the complication rate has to be considered before carrying out treatment. Seven of the 75 surgically treated patients had complications requiring revision surgery. The revisions were necessary due to five peri-implant fractures and two intra-articular screws, which suggests lower complication risk in cases receiving the more conservative approach without osteosynthesis but otherwise non-inferior results – a finding, which was statistically significant. This underscores higher complication risks in cases treated with more sophisticated surgical strategies [25–27].

We only found 3 recurrences in the 75 surgically treated patients. Reasons for this might be the majority of histologically benign lesions in the present study, limited follow-up time, but also surgical strategy. In surgically treated cases, common strategies were performed to reduce the risk for recurrence [28] using rigorous curettage, additional administration of a high-speed burr, the application of H_2_O_2_ and in most cases filling the former tumor cavity with bone-cement, which has the potential added effect of thermal necrosis of remaining tumor cells due to the exothermic polymerization process. The selected surgical treatment approach, involving the extensive intralesional curettage instead of a wider more aggressive resection is a well-reported method, which results in improved functional outcome and sufficient oncologic safety compared to more aggressive surgical strategies [14, 16, 17, 29], which was also supported by our low recurrence rate. We could not find significant differences between the cases treated surgically using bone-cement and cancellous bone, but this may also be attributed to the lower number of cases with cancellous-bone filling.

Interestingly, all recurrences occurred in cases with initial histological diagnosis of benign enchondroma. Of those, two again were histologically classified as enchondroma upon revision. In both cases, it cannot be confirmed that it was in fact a recurrence and not residual tumor. The third recurrence was histologically classified as ACT upon revision. This verifies the evident risk for malignant transformation of initially benign enchondromas with reported risks from 1 to 9%, [1–5] with the need for long term follow-up of aggressive enchondromas. Another explanation might be the known difficulties of histological diagnosis.

We did not find transformation into higher grade chondrosarcomas. Limited follow-up time but also selection of lesions located in the long bones of the extremities without axial and pelvis tumors may explain missing further transformations into higher grade chondrosarcomas [1, 7]. Rigorous follow-up of radiologically aggressive lesions, however, seems crucial to identify a potential transformation into ACT or even higher-grade chondrosarcomas. The present study cannot answer the question as to how often radiological follow-up is required. We generally recommend a first follow-up MRI after 3 to 6 months and then every 12 months thereafter. A problem with the conservative strategy might be patient compliance, which is essential for sufficient oncological safety. Ideally, follow-up should be carried out in specialized orthopaedic oncology departments.

We only analyzed lesions of the long bones, since more distally located enchondromas of the small bones of the hands and feet show histological differences and appear to have a lower potential for malignant transformation or metastasizing compared to lesions in the long bones [30, 31].

No metastases were noted in our collective during the follow-up period of 7 years, however, later metastatic occurrence after this time cannot be ruled out. The risk for later metastases should be extremely low, since pulmonary metastases were only described in ACTs of the long bones after local recurrence and transformation into higher grade (grade II) chondrosarcomas [7, 32]. Based on these experiences, we currently do not recommend regular pulmonary imaging for these benign to low-grade lesions unless we have reasonable suspicion for malignant transformation or metastatic disease.

In 20 cases, we performed an initial incision biopsy prior to intralesional curettage. In all cases, our initial histological results were verified by final histology after intralesional curettage, despite the reported risk for sampling error and underestimation of the degree of dedifferentiation [33, 34]. The only case showing a possible misclassification was the one case with an initial histological classification of enchondroma after incisional biopsy and then recurrence 6 years later showing evidence of ACT. The time-intervals, however, support a later malignant transformation from enchondroma into ACT instead of misdiagnosis in the biopsy. Hence, we still recommend incisional biopsy if there is any doubt regarding malignancy. The present study did not include cases with initial suspicion for higher grade chondrosarcomas (extensive cortical bone enlargement, large interruption of the cortex, extensive invasion of the soft tissues). In such cases, different treatment algorithms are required. Here we always recommend initial biopsy and extralesional tumor resection. For cases with radiological diagnosis of aggressive enchondroma or ACT, where a higher-grade malignancy seems radiographically unlikely, the intralesional curettage strategy is favorable. This is confirmed by the fact that we did not find higher grade chondrosarcomas in the immediately resected lesions in the present study.

It remains unclear, what is the most favorable treatment option for clinically and radiologically aggressive enchondromas and ACTs, since scientific evidence for or against surgery is missing for these benign to low-grade malignant tumors. To the best of our knowledge, this is the first study with such a large patient cohort to evaluate this collective and compare surgical and non-surgical outcome. A potential selection bias of this retrospective study, however, has to be discussed as an important limitation. Decision making towards surgery or conservative follow-up could not be controlled in the present study. This is also reflected in the slightly, but statistically larger lesion sizes of the surgically treated cohort. To minimize the selection bias between the surgery and non-surgery groups, we performed further sub-group analyses. We selected sub-groups with tumor sizes smaller and larger than the mean size of the series (4.4 cm). The sub-group with lesions larger than 4.4 cm offered the best comparability between the surgery and the non-surgery groups, as in these cases lesion size was not significantly different between both groups. Histopathologic differences between the surgery and the non-surgery groups cannot be ruled out as most conservatively treated tumors did not receive a biopsy. The conservatively treated lesions did not show considerable increase in tumor size or progression of scalloping until final follow-up. They may be more comparable to the cases that were surgically treated and histopathologically classified as enchondromas.

Which chondroid lesions clearly need surgery and which do not need surgery, cannot be sufficiently answered by the present study, as we only differentiated the lesions in respect to lesion size and histology. Further subgroups depending on initial clinical appearance and radiological signs of aggressiveness could not be obtained as we did not find significant differences in our highly selected study population. Future studies should also compare subgroups of lesions with considerably different aggressiveness, to achieve objective data on which lesions will benefit from surgery.

This is the first study proving benefits in clinical and functional outcome of cases conservatively followed as opposed to those treated surgically. Sampath et al. only recommend surgical therapy for actively growing lesions with more than 6 mm growth within 3 years [15]. Both good clinical outcome and sufficient safety have been described using this watch-and-wait strategy [4, 9, 17, 18]. From a histological standpoint, surgery would not have been needed to achieve sufficient oncological safety in our collective, since the histology revealed that the majority of our cases were benign enchondromas.

## Conclusion

Our series of conservatively and surgically treated enchondromas and ACTs displayed excellent clinical outcomes. The conservative approach showed a significantly better functional outcome compared to the surgical treatment, and appears to be a sufficiently safe treatment option if strict clinical and radiographic follow-up is assured. For the cases treated surgically, the feasibility of intralesional curettage was confirmed but overall results did not prove superiority compared with only conservative wait and see strategy.

## Abbreviations

ACT: Atypical cartilaginous tumor (chondrosarcoma grade I according to older WHO classification)
MSTS score: Musculoskeletal tumor society score
WHO: World Health Organization
